# Portion Size Norms of Discretionary Foods and Eating Settings: A Repeated Cross-Sectional Study

**DOI:** 10.3390/nu16213670

**Published:** 2024-10-28

**Authors:** Qingzhou Liu, Margaret Allman-Farinelli, Anna Rangan

**Affiliations:** 1 School of Life and Environmental Sciences, Faculty of Science, The University of Sydney, Sydney, NSW 2006, Australia; qingzhou.liu@sydney.edu.au; 2 Charles Perkins Centre, The University of Sydney, Sydney, NSW 2006, Australia; margaret.allman-farinelli@sydney.edu.au; 3 Discipline of Nutrition and Dietetics, Susan Wakil School of Nursing and Midwifery, Faculty of Medicine and Health, The University of Sydney, Sydney, NSW 2006, Australia

**Keywords:** discretionary foods, dietary assessment, consumer behaviors, eating settings, portion sizes

## Abstract

Background: The increase in serving sizes of energy-dense nutrient-poor discretionary foods over time, with attractively presented large servings and package sizes, has led to portion distortion and a new ‘normal’ for serving sizes. Little data exists on the variations of portion size norms of discretionary foods across settings. This study aimed to examine the differences in the range of normal portion sizes of commonly consumed foods between home and out-of-home settings (coffee shops, restaurants). Methods: A repeated cross-sectional design was used, with nine selected discretionary foods and beverages included in a validated online image-series questionnaire. Participants completed the questionnaire at two time points to report their normal portion sizes in home and out-of-home settings. Quantile regression models were used to examine differences in the range of normal portion sizes (17th to 83rd percentile, representing the majority of the study population) between settings. Results: A final sample of 295 participants was included in the analysis (51% females, mean age 40 ± 14 years). The ranges of normal portion sizes did not differ by settings for all test foods except for sugar-sweetened beverages (SSB) tested in both containers and glassware. SSB showed smaller normal portion sizes at home compared to fast food restaurants (in a bottle/can and in a glass/cup; *p* < 0.001). Conclusions: These findings suggest that the portion size norms of many discretionary foods are mostly consistent in home and out-of-home settings. As the typical serving sizes available to consumers in the out-of-home settings are large, it is essential to establish practical serving size guidelines directed at the food industry to increase the availability of smaller size options and empower consumers towards better portion control.

## 1. Introduction

Serving sizes have increased significantly in the last two decades and have been identified as a major contributor to the ‘obesogenic’ food environment that promotes excessive intake of food and energy [1,2]. Large servings and packages are highly accessible and are often more attractively presented in the food environment, with lower unit prices and better value for money [2,3]. Routine exposure to large serving sizes can shift the perceptions of a person’s normal portion size from their regular size towards a larger size, a phenomenon known as portion distortion [4,5]. This is particularly concerning for energy-dense nutrient-poor discretionary foods that are not necessary for a healthy diet [6]. As these larger or super-sized discretionary foods become the new “normal” [4,5,7], this results in passive overconsumption and the risk of overweight and obesity over time [7,8].

Perceptions regarding normal portion sizes have been categorized as one type of portion size norm [9,10,11,12]. This norm is described as a typical perception of how much to consume at a single eating occasion, which plays an important role in the portion size selection process and actual consumption [9,10,11,12]. For example, consumers were found to be more likely to accept small reductions in serving sizes if these were close to their norms, leading to reduced intakes without triggering compensatory eating [9,10,11,13]. In contrast, large reductions in serving sizes (that is, serving sizes considered as “smaller than normal”) can cause additional intake at subsequent occasions [9,10,11,13].

Existing evidence has shown the complexity and variability of portion size norms [4,12]. A wide range of portion sizes could be considered “normal” across individuals, depending on multiple internal and contextual factors [9,10,11,12,13]. The influences of individual characteristics, such as biological sex, weight status, food liking, and physiological factors, such as hunger level, on portion size norms have been consistently demonstrated; males, those classified as obese (BMI over 30 kg/m^2^), those who were more hungry, and those with a higher liking of test foods select larger amounts of foods as their normal portion sizes [10,14,15]. In addition, previous studies have recognized the key role of external eating situations on food intake [16,17]. A large cross-sectional survey in the US showed that consumers tended to have more snacks when they were alone (versus with friends or family) and at home (versus socializing) [16]. Another study found that participants showed better portion control and consumed significantly smaller amounts of provided food in laboratory settings compared with at home in free-living conditions [17]. A potential mechanism may be the desire to follow social expectations and behave in a socially acceptable manner [18,19]. People tend to monitor their intake closely to avoid negative judgement, especially for discretionary foods that are expected to be consumed in small amounts [18,20,21].

Nevertheless, there is a lack of research exploring the potential influence of contextual factors on portion size norms [12]. In Australia, the Industry Guide to Voluntary Serving Size Reduction (Industry Guide) has been developed to support the food industry in reducing the serving sizes of discretionary foods [22]. The Industry Guide incorporates retail and out-of-home settings, with maximum serving size recommendations tailored for these settings (that is, the maximum limits for serving size options) [22]. These recommendations were directed at the food industry to reduce the availability of super-sized options and encourage the introduction of smaller serving size options below the maximum limits [22]. However, the feasibility of the implementation of the Industry Guide is unknown; it is unclear how portion size norms vary by eating settings, and whether the maximum serving size recommendations align with Australian consumers’ portion size norms. Individual factors that influence portion size norms have been reported in the authors’ previous work [23]. The present study explicitly focused on the influences of eating settings on normal portion sizes to gain a more comprehensive understanding of the conceptualization of portion size norms. This will help inform the development of tailored nutrition education and serving size recommendations for Australian consumers [1,12,24].

Therefore, the primary aim of this study was to investigate the effect of eating settings on the portion size norms of commonly consumed discretionary foods and beverages among Australian consumers; this was achieved by comparing the selected normal portion sizes at home and in out-of-home settings (coffee shops, fast food restaurants). The secondary aim was to compare the ranges of normal portion sizes to the maximum serving size recommendations outlined in the Industry Guide.

## 2. Materials and Methods

### 2.1. Study Design

A repeated cross-sectional design was used to examine the range of normal portion sizes of commonly consumed discretionary foods. Participants were required to complete the online survey twice, at least one week apart (November 2022 to January 2023) to provide a more accurate estimate of their normal portion size, given the number of potential influencing factors [4,12]. The survey was developed using Qualtrics, consisting of demographic questions and an image-series questionnaire to assess the normal and perceived appropriate portion sizes. The demographic questions collected information on participants’ biological sex, age, postcode of home address, self-reported height and body weight, usual physical activity level (PAL), education level, food literacy and hunger level. Based on self-reported residential postcodes, the socio-economic indexes for areas (SEIFAs) were used to assign participants into deciles (1–10) of economic disadvantage [25], with deciles 1–5 grouped as lower socioeconomic status (SES) and deciles 6–10 as higher SES. Baseline hunger level was assessed using a validated visual analogue scale of 0–100, with 0 indicating “not hungry at all” and 100 “extremely hungry” [26,27]. PAL was estimated based on the frequency and intensity of usual exercise and was classified into four categories: sedentary, lightly active, moderately active, and very-to-extremely active [28,29]. As a marker of food literacy, cooking confidence was assessed using a 5-point validated Likert scale (can cook a nutritious meal; can cook a meal in a short amount of time; can cook without spending a lot of money; can follow a recipe) [30,31]; a total score equal or higher than 16 out of 20 was classified as a high cooking confidence; otherwise, it was classified as low [30,31].

The image-series questionnaire used in the survey was previously validated in the population of interest in an in-person laboratory session, in which participants reported their normal portion sizes twice based on the image-series and equivalent real-food portion size options [32]. This online image series showed good agreement, with an overall intraclass correlation coefficient of 0.85, for images compared with real foods [32], and thus it was considered suitable to be used in the present study with a more representative sample of Australian consumers.

In this study, nine discretionary foods and beverages were included according to the latest national data [29,30]. These foods were the most frequently consumed by the Australian population and are readily available in Australian retail stores and outlets [29,30]. These included four cake types (layered cake, caramel slices, muffins, and banana bread) as mid-meal snacks, three fast foods as meals (pizza) or side dishes (hot chips and nuggets), and sugar-sweetened beverages (SSB; cola, in glasses or cups, and in bottles or cans) as between-meal drinks. Settings were at home versus out of home at coffee shops (for cakes) or fast food restaurants (for fast foods and SSB).

**Figure 1:**
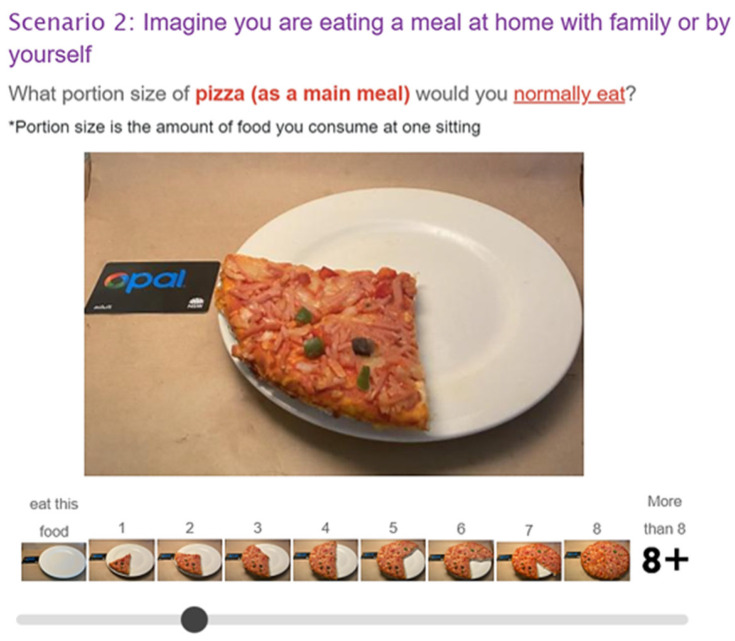
Survey question example. The eight successive images corresponding to the sliding scale question, labelled from smallest ‘1’ to largest ‘8’, and additional selections of ‘0—I do not eat this food’ and 9—greater than the largest option displayed’. Participants were instructed to select the closest match to their normal portion sizes based on image options.

For each test food, eight successive images of portion size options were displayed, corresponding to the sliding scale question, “What portion size of (the test food, for example, pizza as a meal) would you normally eat (the context, for example, if you are at home with family or by yourself; if you are eating out with a friend)?” The definition of portion size as ‘the amount of food you consume at one sitting’ was displayed for each question. The sliding scale was numbered from smallest ‘1’ to largest ‘8’, with the additional selections of ‘0—I do not eat this food’ and ‘9—greater than the largest option displayed’. A forced response was set for each question; participants were required to move the cursor to make a selection before proceeding to the next question. When participants moved the cursor along the scale to their corresponding or nearest selection, the selected images would become enlarged for easier viewing (Figure 1). Details of the survey questions and portion size options are presented as Supplementary Materials (Tables S1 and S2).

### 2.2. Recruitment and Study Procedure

The following eligibility criteria applied to this study: aged between 18–65 years, living in Australia, fluent in English, and no current or previous diagnosis of an eating disorder. According to reviews of previous dietary assessment studies [10,11,33,34,35], a quota of 50 for each sex and age group (18–30, 31–50, 51–65 years) was set. A target sample size of 300 participants from the general Australian population was deemed sufficient to answer the research question.

Participants were recruited through the distribution of physical flyers in local communities and around the university and online advertisements via social media sites. Potential participants were directed to an online screening questionnaire; those who passed the screener and provided informed consent were then able to proceed to the demographic questionnaire that was built into the first survey, followed by the image-series questionnaire to assess the normal portion sizes. The second survey was sent to participants one week after completing the first survey; participants’ answers between the two surveys were linked in an anonymous manner by creating a username.

This study was approved by the Human Ethics Committee of the University of Sydney (ethics approval number 2022/147). Participants received a small compensation voucher ($10 AUD supermarket voucher) after completing both surveys.

### 2.3. Data Quality Check and Analysis

The quality of collected data was carefully checked considering that the study was conducted online with self-reporting by participants. To maintain data quality, incomplete surveys, repeated responses, and surveys completed by bots were detected and excluded (n = 10) using an embedded function in Qualtrics [36]. The responses at both time points were manually checked by the research team to exclude participants who selected the same portion size options throughout the survey (for example, if a participant selected the middle option for all questions); however, no response required exclusion.

Descriptive analyses were performed using IBM SPSS v28 (IBM, Armonk, NY, USA, 2021) to describe participant characteristics. For each food, data were excluded if participants reported not consuming a particular food or drink item in both surveys. Participants who responded as “other” or “prefer not to say” for sex (n = 3) were excluded from the final sample for data analysis. Including such a small subgroup could lead to inaccuracy in model estimates due to insufficient statistical power, and this could affect the overall robustness of the analysis [37].

Given that previous evidence showed that a large range of portion sizes could be considered normal across individuals and the conceptualization of portion size norms is affected by multiple influencing factors [9,13], the 17th and 83rd percentiles were selected to represent the lower and the upper boundaries of the range of normal portion sizes. This range included portion size selections of a clear majority (two thirds) of the study population. Quantile regression analysis was conducted to assess the differences in normal portion sizes between settings while adjusting for potential influencing factors. A quantile regression model was established per test food, resulting in nine models in total. The input variables were the portion size image options selected by participants (between 0–9). The output variables were the predicted values after adjusting for potential influencing factors, and the significance and effect sizes (coefficients; representing the change in the output variable for a one-unit change in the influencing factor) at the 17th and 83rd percentile.

Within the model, the data from both survey times were included, and participant ID was used as a random effect to account for repeated measures per participant. The measures of normal portion sizes in different settings (depending on food types; home versus coffee shops or home versus fast food restaurants) were included as a fixed factor to assess the differences. The fixed effects of other potential influencing factors, including sex, age groups (18–30, 31–50, 51–65 years), BMI (calculated from height and weight data, in kg/m^2^), PAL (four categories), food literacy (the total score), baseline hunger level at two time points (visual analogue scale 0–100), and SES (deciles 0–10), were also adjusted in the model. All quantile regression models were performed using “lqmm” package in R statistical software version 2023.10.30 (R Core Team, Vienna, Austria, 2023) [38]. *p*-values were adjusted for multiple comparisons using the Holm’s sequential Bonferroni procedure [39].

## 3. Results

### 3.1. Participants Characteristics

Out of 692 participants who passed the screening questionnaire and provided informed consent, a final sample of 295 participants completed both surveys and were included for data analysis (51% females, mean age 40 ± SD 14 years). For baseline hunger levels, most participants reported not feeling hungry at both time points (median hunger 27 of 100 in the first survey, 28 of 100 in the second survey). Details of participants’ characteristics and demographic information are present in Table 1.

### 3.2. The Range of Normal Portion Sizes and Differences by Settings

The range of normal portion sizes (lower boundary at 17th percentile, upper boundary at 83rd percentile) at home and in out-of-home settings is presented in Table 2. Across the four cake types, the range of normal portion sizes for banana bread was the largest (home 95–148 g; café 91–141 g), followed by muffin (home 56–122 g; café 67–124 g), layered cake (home 94–120 g; café 95–120 g), and caramel slices (home 49–66 g; café 50–67 g). For fast foods, the range of normal portion sizes for pizza as a main meal (home 146–259 g; restaurant 144–257 g) was larger than hot chips (home 108–175 g; restaurant 106–170 g) and nuggets (home 104–151 g; restaurant 101–148 g) as side dishes. The range of normal portion sizes for SSB in cup/glass (home 266–414 mL; restaurant 285–433 mL) was approximately 40 mL larger than that for SSB in bottles/cans (home 230–359 mL; restaurant 250–400 mL).

The differences in the range of normal portion sizes between home and out-of-home settings were tested (Table 2). Overall, the ranges of normal portion sizes between settings were not significantly different for seven out of nine test foods; the effect sizes were small and varied from −0.13 to 0.05 across food types, with less than 10 g differences between settings noted.

Significant differences between settings were only observed for SSB in cups/glasses and in bottles/cans, with normal portion size ranges being larger at fast food restaurants compared with at home (*p* < 0.001). The effect sizes varied from 0.26 to 0.33, indicating that participants’ normal portion sizes of SSB were approximately one third an option (20–40 mL) smaller at home compared with at fast food restaurants.

### 3.3. Comparison of Normal Portion Sizes to Maximum Serving Size Recommendations

**Figure 2:**
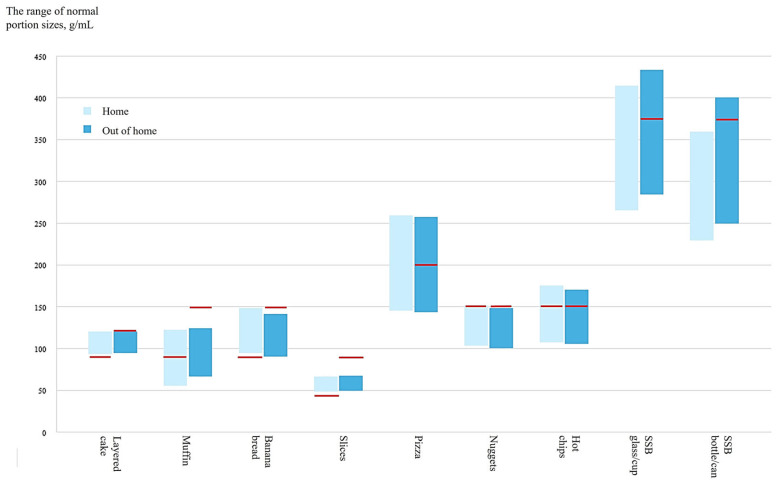
The range of normal portion sizes selected by participants (17th–83rd percentiles) compared to the maximum recommended serving sizes, by food type. Upper and lower boundaries of the box indicate quantile regression outputs at 17th and 83rd percentiles, respectively. Red lines indicate the maximum serving size recommendations from the Industry Guide to Voluntary Serving Size Reduction [22]. For SSB in glasses/cups and in bottles/cans, recommendations for a medium-size meal (375 mL) and a large-size meal (450 mL) were both included. No recommendation is available for pizza, SSB glasses, and SSB bottles in home settings, and thus these are not shown on this figure.

The range of normal portion sizes selected by participants for home and out-of-home settings (coffee shops, fast food restaurants) was compared with the maximum serving size recommendations from the Industry Guide for retail (typically consumed at home) and out-of-home settings, respectively (Figure 2) [22].

In the home setting, both the lower and upper boundaries of normal portion size ranges for layered cake, banana bread, and slices were above the recommended serving sizes (90 g, 90 g, and 45 g, respectively). This indicated that most participants had a normal portion size larger than the recommendations. For muffin, approximately half of participants selected normal portion sizes that were larger than the recommended serving sizes (90 g). This was similar for hot chips, with approximately half of participants reporting normal portion sizes larger than the recommended serving sizes (150 g). For nuggets, the normal portion size range was below the recommended serving sizes (150 g). As there are no current recommendations for pizza or SSB purchased in the retail setting, these foods could not be compared.

In out-of-home settings, the normal portion sizes selected by most participants were below the maximum serving sizes of discretionary foods set in the Industry Guide. In coffee shop settings, the ranges of normal portion sizes for all four cake types were below the maximum recommended serving sizes (90 g for slices, 150 g for other cake types). Similarly, in fast food restaurants, most participants selected a normal portion size of nuggets below the maximum recommendation (150 g), but for pizza and hot chips, the upper boundary of the normal portion size ranges exceeded the maximum recommended serving sizes (257 g vs. 200 g, 170 g vs. 150 g, respectively). For SSB in glasses/cups and SSB in bottles/cans, the upper boundary of the normal portion size ranges was above the recommendations for a medium-sized meal (375 mL) but was below those for a large meal (450 mL) as set in the Industry Guide.

## 4. Discussion

This study aimed to investigate the portion size norms of discretionary foods and beverages as consumed at home and in out-of-home settings by comparing the ranges of normal portion sizes of nine test foods between home versus out-of-home settings in coffee shops or fast food restaurants. Overall, there were few differences in the ranges of normal portion sizes between settings. Consumers’ perceptions of their normal portion sizes appeared to be similar at home when eating alone or with family and out of home with friends. A potential explanation could be that the conceptualization of normal portion sizes is primarily based on personal norms (that is, how much individuals would consume at one eating occasion according to themselves) rather than social norms (that is, how much individuals believe other people expect them to eat at one eating occasion) [10,21,41]. Evidence suggests that personal norms originate from individuals’ conscious reasoning and pre-existing attitudes [41]. For portion sizes, personal norms are established based on individuals’ prior experiences and eating habits [21,42]. This may be especially true for foods that are sold in food units, with the number of food units being used as an informative cue to help make portion size decisions (for example, one muffin as a mid-meal snack, three chicken nuggets as a side dish) [43]. The exception was SSB, where the range of normal portion sizes was significantly larger at fast food restaurants compared to at home, although the magnitude of differences in actual volume was less than 40 mL and considered small. This was true regardless of whether SSB was consumed in a glass/cup or in a can/bottle.

The difference in SSB portion size norms between settings is consistent with other studies examining the effect of contextual factors on SSB intake among Australian consumers; larger amounts of SSB were consumed at social settings (restaurants with friends) compared with consumption at home [44,45]. This pattern was also observed for solid foods, but it appeared to be subject to co-eater familiarity, and contradictory results were noted across studies [11,14,46]. Eating socially with friends was linked to increased food consumption compared with eating alone [11,14,46]. When eating among unfamiliar strangers, however, people tended to select smaller meals and consume less to create a positive impression of being healthier [46,47]. The finding in this study suggests that the increased intake of SSB when dining out with friends may have become a norm. Additional studies are required to confirm this finding and better understand the mechanism underlying the inconsistencies in beverage portion size norms across settings and eating contexts as well as the potential consequences on total energy intake over the long term.

For most selected foods, the range of normal portion sizes selected by Australian consumers was lower than the maximum recommendations set in the Industry Guide [22]. It must be acknowledged that the maximum serving sizes are not necessarily recommended serving sizes, they are merely upper limits that should not be exceeded when serving foods and beverages [22,48]. The study’s findings suggest that the majority of consumers are likely to regard the maximum recommended serving sizes as acceptable. Considering the ubiquity of large serving sizes, especially in out-of-home settings [1,2], there may be a misalignment between consumers’ portion size norms and available serving size options. This highlights the potential opportunity for the food industry, business owners, and retailers to reduce the availability of supersized options and introduce smaller serving sizes that better align with the recommendations and consumers’ portion size norms [1,5]. However, for cakes consumed in home settings (purchased in a retail setting such as supermarkets), the range of normal portion sizes exceeded the maximum recommended serving sizes. A previous audit study showed that the serving sizes of cakes sold in Australian coffee chains (median serving size 148 g) were significantly larger than supermarket equivalents (median serving size 58 g) [49]. Consumers may regard the single serving size of cakes sold in Australian supermarkets that meet the Industry Guide maximum recommendations as being “too small”, as they do not align with their norm [22,49]. In order to prevent further portion distortion and associated negative health consequences, such as excessive energy intake and increased risks of obesity and non-communicable diseases, relevant public health recommendations are required to inform consumers about the appropriate portion sizes for different eating situations [1,7,50,51].

There are no official guidelines regarding serving size suggestions for discretionary foods. The current serving size labelling on the nutrition information panel (NIP) of packaged foods has been found to be highly variable across and within products, as these amounts are determined by the food manufacturer [6,52,53]. For instance, the declared serving size on the NIP of SSB can vary from 100 mL to 700 mL [52]. The 600 kJ standard serve of discretionary food from the Australian Dietary guidelines is not a practical recommendation for how much to consume in one sitting, and the variations in food types and eating occasions were not considered (for example, a chocolate bar as a mid-meal snack versus pizza as a main meal) [6]. In addition, the current policies on serving and portion sizes primarily rely on voluntary initiatives. One of these initiatives was the Industry Guide from the Healthy Food Partnership, which aimed to set a cap for the food industry to reduce the availability of super-sized options [22], whereas the uptake rate of this initiative is currently unknown. Other challenges in the food environment should also be recognized [54,55]. For instance, the value size pricing strategy is used by the food industry; that is, discretionary foods and beverages in larger serving sizes are more heavily promoted and offer better value for money compared with smaller sizes. To help consumers in monitoring their intake and selecting appropriate portion sizes based on their needs, guidance from public health authorities and government directed at the food industry sector is required [1,56]. The data collected on normal portion sizes in this study, along with other potential influencing factors, such as the availability and cost of serving size options, sale and marketing strategy, and the occurrence of shrinkflation (that is, reducing the serving or package sizes while maintaining the same price), should all be carefully considered in the development of pragmatic serving size guidelines across discretionary food categories [52,53,57].

It should be acknowledged that portion size norms represent what people consider as their normal portion sizes, and thus they differ from typical intakes derived from national nutrition surveys (that is, the median portion sizes consumed per eating occasion). The normal portion sizes for six out of eight test foods were smaller than the reported portion sizes from the latest national nutrition survey (there were no data for nuggets, so these could not be compared) [58]. For example, the ranges of normal portion sizes for SSB at home and in out-of-home settings were 230–414 mL and 250–433 mL, respectively, whilst the median portion sizes from the national survey were close to the upper boundaries of these ranges, with 364 mL for females and 390 mL for males [58]. It may be that the conceptualization of normal portion sizes did not take into account other cues embedded within the food environment, such as the tendency to consume everything on one’s plate due to concerns about wasting food [59,60]. The robust portion size effect might be another potential explanation, that is, larger serving sizes lead to increased consumption without consumers’ awareness [2,61]. This indicates that in real life situations, following the perceptions of normal portion sizes can be challenging, especially when the serving size is large, as individuals may consume more than intended [62]. This finding is in line with previous qualitative studies [3,63]; people acknowledged that the variation in actual portion size decisions were dependent on the contextual situation, with some self-restricting portion sizes or, conversely, consuming a larger amount to impress people. Consumers reported that the difficulties in portion control were due to various reasons, including a lack of their preferred serving size options, large serving sizes being served, distractions, and social pressure [3,23,63]. Therefore, changes to the broad food environment are crucial to facilitate the reduction of both portion size norms and actual consumption [1,64]. More effective public health initiatives that actively engage the food industry, public health organizations, and policy makers are needed to make positive changes to the food environment to facilitate better portion control [1,65,66]. For example, using on-pack visual cues or resealable packages, clearer serving size labels, and providing a wider range of smaller serving and package size options with proportional pricing are some of the strategies that could be explored [66,67]. Future research should be conducted to understand consumers’ and other relevant stakeholders’ attitudes toward these potential public health interventions targeting portion control.

### Strength and Limitations

This study has several strengths. The online design allowed the inclusion of a large sample of consumers from all over Australia, and potential influencing factors were controlled within the quantile regression models. The image-based questionnaire was tested against real food portion size options and found to have good validity in the target population [32], with the eating setting and presence of other people (for example, with family) clearly described to avoid confusion. The BMI profile of the recruited sample, with two thirds of participants classified in the overweight and obese category, is considered representative of the Australian population [68].

However, limitations of this study should be acknowledged. Most participants were highly educated and from higher SES areas, which may not be representative of the general Australian population. The chance of estimation bias exists, as participants were required to conceptualize their normal portion sizes according to past experience [32]; accurate estimations of portion sizes can be challenging, especially given the wide variations in tableware sizes and default serving sizes for home and out-of-home settings in real life [69,70]. Although a representative range of serving size options was included, some common serving or package sizes (for instance, fast foods in takeaway containers) were not covered in the current study, and only two out-of-home settings were selected to reduce participant burden. We acknowledge the presence of various other influencing factors within the broad food environment (for instance, sociocultural variables such as ethnicity and cultural traditions), and thus the obtained data on normal portion sizes require careful interpretation [71,72,73,74]. Further studies should include additional contextual factors influencing eating behaviors, with a more detailed description of the presence of other people and relationships (for example, family gathering versus social gathering versus unfamiliar strangers) as well as cultural, financial and emotional contexts for a clearer understanding of the influence of contextual factors on the conceptualization of portion size norms [75]. Additional research performed in real-life settings is needed to address the uncertainties around when and how individuals follow their perceptions of normal portion sizes during serving size selections at the point-of-purchase and subsequent portion size decisions and consumption.

## 5. Conclusions

This study compared the range of normal portion sizes for home and out-of-home settings for selected discretionary foods and beverages. There were no significant differences between settings for all discretionary foods except SSB, suggesting that the portion size norms of many discretionary foods are consistent in home and out-of-home settings. If confirmed, this shows a misalignment between the portion size norms of consumers and the ubiquity of large serving sizes available in the out-of-home settings, potentially contributing to further portion distortion. More efforts are required from public health authorities and the government to target portion distortion by the food industry. These may include increasing the availability of smaller size options, as well as establishing clearer and more consistent guidance to empower consumers towards more appropriate portion size selections.

## Figures and Tables

**Table 1:** Participants’ characteristics (n = 295).

Age, years, mean (SD)	40 (14)
18–30, n (%)	98 (33)
31–50, n (%)	99 (334)
51–65, n (%)	98 (33)
Sex, males, n (%)	145 (49)
BMI ^a^, kg/m^2^, mean (SD)	27 (7)
Underweight, n (%)	8 (3)
Within normal weight range, n (%)	124 (42)
Overweight, n (%)	91 (31)
Obese, n (%)	72 (24)
Education level, n (%)	
High school or below	55 (19)
Diploma equivalent	49 (17)
University	191 (75)
Socio-economic status (SES) ^b^, n (%)	
Lower	67 (23)
Higher	221 (77)
Physical activity level (PAL) ^c^, n (%)	
Sedentary	55 (19)
Lightly active	137 (46)
Moderately active	82 (28)
Very-to-extremely active	21 (7)
Food literacy ^d^, n (%)	
Low	112 (38)
High	183 (62)

^a^ BMI (kg/m^2^): less than 18.5 is underweight, 18.5–24.9 within normal weight range, 25–29.9 overweight, >30 obese [40]. ^b^ SES: the socio-economic indexes for areas (SEIFAs) were used to assign participants into deciles (1–10) of economic disadvantage based on self-reported residential postcodes [25]; deciles 1–5 were grouped as lower socioeconomic status (SES) and deciles 6–10 as higher SES. ^c^ PAL: estimated based on the frequency and intensity of usual exercise, and classified into four categories, which are sedentary, lightly active, moderately active, and very-to-extremely active [28,29]. ^d^ Food literacy: as a marker of food literacy, cooking confidence was measured using a 5-point validated Likert scale (can cook a nutritious meal; can cook a meal in a short amount of time; can cook without spending a lot of money; can follow a recipe) [30,31]; a total score equal or higher than 16 out of 20 was classified as high cooking confidence, otherwise, it was classified as low [30,31].

**Table 2:** The range of normal portion sizes between settings, by food type (n = 9).

Test Food	Range of Normal Portion Sizes		Effect Sizes ^a^	*p*-Values ^b^
	17th–83rd	17th–83rd		
	Home	Coffee shops		
Banana bread	95–148	91–141	−0.13	0.02
Caramel slices	49–66	50–67	0.05	0.52
Layered cake	94–120	95–120	0.01	0.87
Muffin	56–122	67–124	0.03	0.53
	Home	Fast food restaurants		
Pizza	146–259	144–257	−0.03	0.49
Hot chips	108–175	106–170	−0.10	0.11
Nugget	104–151	101–148	−0.06	0.26
SSB ^c^ cup/glass	266–414	285–433	0.26	0.005 *
SSB bottle/can	230–359	250–400	0.33	<0.001 *

^a^ The effect sizes are the coefficients from quantile regression models at percentiles tau = 0.17 and 0.83. Differences in normal portion sizes between settings were tested in the models as fixed factors. ^b^ *p*-values adjusted for multiple comparisons using Holm–Bonferroni method [39], * *p*-values were statistically significant after the adjustment. ^c^ SSB: sugar-sweetened beverages.

## Data Availability

The data presented in this study are available on request from the corresponding author due to ethical restrictions.

## Supplementary Materials

The following supporting information can be downloaded at: https://www.mdpi.com/article/10.3390/nu16213670/s1: Table S1. Survey questions for home and out-of-home settings, by food types (n = 9). Table S2. Portion size weights of discretionary foods and beverages included in this study (n = 9), in gram or milliliter.
