# Supplementary material for: Portion Size Norms of Discretionary Foods and Eating Settings: A Repeated Cross-Sectional Study
Source: Nutrients. 2024 Oct 28;16(21):3670. doi: 10.3390/nu16213670 (PMC11547615; doi:10.3390/nu16213670)
Supplement: Supplementary file 1 [file nutrients-16-03670-s001.zip › nutrients-3231086-supplementary.pdf]

Supplementary materials

Table S1. Survey questions for home and out of home settings, by food types (n=9).

**Test foods**	**Survey questions**	
	Home	Out of home
**Cakes**		
Banana bread	Imagine you are having some snack at home by yourself, what portion size of banana breads or similar baked goods as a mid-meal snack would you normally eat?	Imagine you are at café with a friend having a snack, what portion size of banana breads or similar baked goods as a mid-meal snack would you normally eat?
Caramel slices	Imagine you are having some snack at home by yourself, what portion size of caramel slices or similar as a mid-meal snack would you normally eat?	Imagine you are at café with a friend having a snack, what portion size of caramel slices or similar as a mid-meal snack would you normally eat?
Layered cakes	Imagine you are having some snack at home by yourself, what portion size of cakes as a mid-meal snack would you normally eat?	Imagine you are at café with a friend having a snack, what portion size of cakes as a mid-meal snack would you normally eat?
Muffins	Imagine you are having some snack at home by yourself, what portion size of muffins or similar as a mid-meal snack would you normally eat?	Imagine you are at café with a friend having a snack, what portion size of muffins or similar as a mid-meal snack would you normally eat?
**Fast foods**		
Pizza	Imagine you are eating a meal at home with family or by yourself, what portion size of pizza as a main-meal would you normally eat?	Imagine you eating out with a friend, what portion size of pizza as a main-meal would you normally eat?
Hot chips	Imagine you are eating a meal at home with family or by yourself, what portion size of hot chips as a side dish would you normally eat?	Imagine you eating out with a friend, what portion size of hot chips as a side dish would you normally eat?
Nuggets	Imagine you are eating a meal at home with family or by yourself, what portion size of nuggets as a side dish would you normally eat?	Imagine you eating out with a friend, what portion size of nuggets as a side dish would you normally eat?
**Sugar-sweetened beverages (SSB)**		
SSB in cup/glass	Imagine you are having some snack at home by yourself, what portion sizes of SSB (from cup/glass, between meals) would you normally have?	Imagine you eating out with a friend, what portion size of SSB (from cup/glass, between meals) would you normally have?
SSB in bottle/can	Imagine you are having some snack at home by yourself, what portion sizes of SSB (from bottle/can, between meals) would you normally have?	Imagine you eating out with a friend, what portion size of SSB (from bottle/can, between meals) would you normally have?

Table S2. Portion size weights of discretionary foods and beverages included in this study (n=9), in gram or millilitre.

	**1**	**2**	**3**	**4**	**5**	**6**	**7**	**8**
**Banana bread**	31	67	100	155	184	221	252	290
**Caramel slices**	20	33	46	67	100	142	168	210
**Layered cakes**	34	65	100	127	170	228	286	374
**Muffin**	12	22	53	105	152	196	230	305
**Pizza**	62	118	182	247	316	374	442	503
**Hot chips**	25	50	75	100	130	170	221	287
**Nugget**	20	60	120	160	240	320	400	480
**SSB in cup/glass**	75	150	225	300	375	450	525	600
**SSB in bottle/can**	125	188	250	300	375	600	-	-
